# Evidence-based Emergency Tracheal Intubation

**DOI:** 10.1164/rccm.202411-2165CI

**Published:** 2025-04-16

**Authors:** Stephanie C. DeMasi, Jonathan D. Casey, Matthew W. Semler

**Affiliations:** ^1^ Department of Emergency Medicine and; ^2^ Division of Allergy, Pulmonary, and Critical Care Medicine, Vanderbilt University Medical Center, Nashville, Tennessee; and; ^3^ Center for Learning Healthcare, Vanderbilt Institute for Clinical and Translational Research, Nashville, Tennessee

**Keywords:** emergency tracheal intubation, preoxygenation, induction medication, laryngoscopy

## Abstract

Millions of critically ill adults undergo tracheal intubation in an emergency department or ICU each year, nearly 40% of whom experience hypoxemia, hypotension, or cardiac arrest during the procedure. Over the last two decades, a series of randomized trials have examined which of the tools, techniques, devices, and drugs used to perform emergency tracheal intubation improve outcomes and which are ineffective or harmful. Results of these trials have demonstrated that preoxygenation with noninvasive ventilation and administration of positive pressure ventilation between induction and laryngoscopy prevent hypoxemia during intubation, video laryngoscopy facilitates successful intubation on the first attempt and may prevent esophageal intubation, use of a stylet is superior to intubation with an endotracheal tube alone and is comparable with use of a bougie, and administration of a fluid bolus before induction does not prevent hypotension. Many additional decisions clinicians face during emergency tracheal intubation are not yet informed by rigorous evidence. Randomized trials must continue to examine systematically each aspect of this common and high-risk procedure to improve patient outcomes and bring forth an era of evidence-based emergency tracheal intubation.

More than 1.5 million critically ill adults undergo tracheal intubation each year in the United States (1). Unlike tracheal intubation in the operating room before elective surgery, during which serious complications are rare, nearly 40% of tracheal intubations in the emergency department (ED) or ICU are complicated by hypoxemia, hypotension, or cardiac arrest, making tracheal intubation one of the highest-risk periods of a patient’s critical illness (2). During each emergency tracheal intubation, clinicians must select between available *1*) approaches to oxygenation and ventilation; *2*) medications for sedation, paralysis, and hemodynamic support; and *3*) devices for laryngoscopy and intubation of the trachea, each of which may affect the likelihood of successful intubation on the first attempt and the risk of serious complications (Figure 1). Over the last 25 years, a series of randomized trials have provided rigorous evidence that directly informs the optimal approach to many aspects of tracheal intubation of critically ill adults. This review summarizes what the evidence from randomized trials tells us about how best to intubate a critically ill adult in clinical care today and what knowledge gaps remain. This review does not address the many aspects of emergency tracheal intubation for which evidence from randomized trials is not yet available to complement clinical knowledge of anatomy and physiology, expert opinion, clinician judgment, observational studies, and extrapolation from the operating room. Many of these topics, such as the anatomically difficult airway, the physiologically difficult airway, awake intubation, and crisis resource management, have been well summarized in previous reviews and guidelines (3–8).

**Figure 1.**
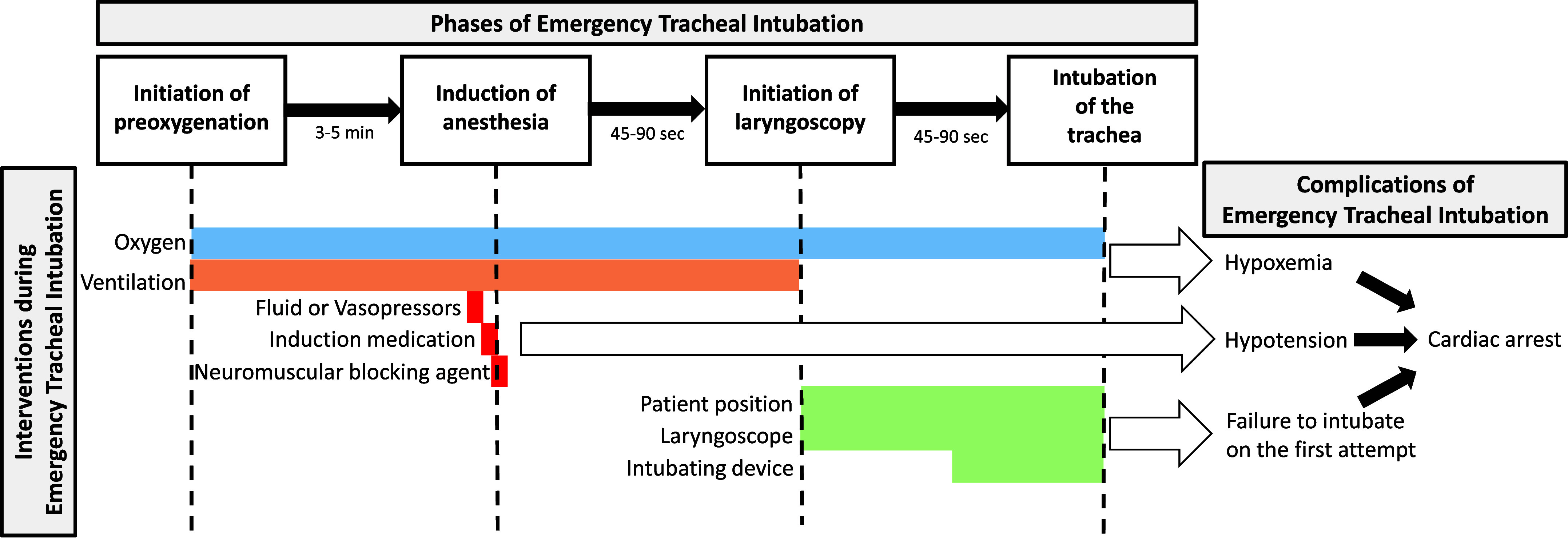
Clinical decisions in each phase of emergency tracheal intubation may affect procedural and patient-centered outcomes. During the three phases of emergency tracheal intubation (preoxygenation, induction of anesthesia to initiation of laryngoscopy, and laryngoscopy and intubation of the trachea), clinicians make numerous treatment decisions regarding *1*) the approach to administration of supplemental oxygen and positive pressure ventilation; *2*) medications related to induction of anesthesia, neuromuscular blockade, and hemodynamic support; and *3*) the tools and approach to laryngoscopy and intubation of the trachea. Conceptually, these choices may affect procedural outcomes (e.g., hypoxemia, hypotension, and failure to intubate on the first attempt), downstream short-term clinical outcomes (e.g., cardiac arrest), or long-term patient-centered outcomes (e.g., death, post-traumatic stress disorder). In the last two decades, how treatment choices during emergency tracheal intubation affect procedural and patient outcomes has been the focus of a series of randomized trials.

## Oxygenation and Ventilation

Hypoxemia occurs during 10–25% of all emergency tracheal intubations (9, 10) and a higher percentage of intubations for acute hypoxemic respiratory failure (2, 11, 12). Hypoxemia during intubation increases the risk of cardiac arrest and death (11, 12). Hypoxemia during emergency tracheal intubation occurs because of factors related to the underlying critical illness (e.g., pulmonary shunt, ventilation-perfusion mismatch, increased oxygen consumption), factors related to the intubation procedure (e.g., apnea from sedation and paralysis, decreased residual capacity from supine positioning), and factors related to the circumstances of the procedure (e.g., limited time for preparation, longer procedural duration, and greater number of intubation attempts). To understand which interventions prevent hypoxemia during intubation, recent randomized trials have examined different approaches to supplemental oxygen administration and positive pressure ventilation during the three phases of the intubation procedure: *1*) before induction of anesthesia (preoxygenation), *2*) between induction of anesthesia and initiation of laryngoscopy, and *3*) between initiation of laryngoscopy and intubation of the trachea.

### Before Induction (Preoxygenation)

Preoxygenation is the administration of supplemental oxygen before induction of anesthesia (Figure 1). By increasing the content and volume of oxygen in the lung at induction, preoxygenation decreases the risk of hypoxemia during the tracheal intubation procedure. Critically ill adults may receive preoxygenation with supplemental oxygen without positive pressure ventilation (i.e., a standard nasal cannula, a high-flow nasal cannula, or an oxygen mask) or with supplemental oxygen and positive pressure ventilation (i.e., noninvasive ventilation or bag mask ventilation). Three randomized trials have found the incidence of hypoxemia during intubation of critically ill adults to be lower when preoxygenation is performed with noninvasive ventilation as compared with an oxygen mask (12–14). The largest of these, the recent PREOXI (Pragmatic Trial Examining Oxygenation Prior to Intubation) trial, found that noninvasive ventilation reduced the incidence of hypoxemia from 18.5% to 8.1% (absolute risk difference, −9.4 percentage points; 95% confidence interval [CI], −13.2 to −5.6) and reduced the incidence of cardiac arrest from 1.1% to 0.2% (absolute difference, −0.9 percentage points; 95% CI, −1.8 to −0.1). Aspiration occurred in six patients (0.9%) in the noninvasive ventilation group and nine patients (1.4%) in the oxygen mask group. Noninvasive ventilation appeared to decrease the incidence of hypoxemia in all subgroups, including patients undergoing intubation for encephalopathy and patients who were not requiring any supplemental oxygen in the hour before enrollment.

High-flow nasal cannula is a potential alternative to noninvasive ventilation for preoxygenation of critically ill adults. Whether using the high-flow nasal cannula for preoxygenation of critically ill adults prevents hypoxemia during intubation of critically ill adults compared with use of either noninvasive ventilation or an oxygen mask remains uncertain. Multiple moderate-sized randomized trials compared preoxygenation with the high-flow nasal cannula with preoxygenation with an oxygen mask among critically ill adults, with inconclusive results (15–19). The one randomized trial to date that directly compared preoxygenation with noninvasive ventilation versus the high-flow nasal cannula among critically ill adults found that hypoxemia occurred in 23% of patients in the noninvasive ventilation group and 27% of patients in the high-flow nasal cannula (absolute difference, −4.2 percentage points; 95% CI, −13.7 to 5.5). Among the subgroup of patients with moderate to severe hypoxemia before intubation, the incidence of hypoxemia was 24% in the noninvasive ventilation group and 35% in the high-flow nasal cannula group (95% CI, −22.3 to 0.3; *P* = 0.053).

Together, these results suggest that most patients undergoing tracheal intubation in the ED or ICU should receive preoxygenation with noninvasive ventilation with either a dedicated noninvasive ventilator (i.e., a bilevel positive inspiratory pressure machine) or a tight-fitting mask attached to a conventional invasive mechanical ventilator. Patients with a clear contraindication to noninvasive ventilation should receive preoxygenation with a high-flow nasal cannula, an oxygen mask, or both.

### Between Induction and Laryngoscopy

For nearly 50 years, clinicians debated whether to administer or avoid positive pressure ventilation during the 45- to 90-second interval between the administration of sedation and neuromuscular blockade and the point at which the medications have taken effect and laryngoscopy can be initiated (20–22). Whether positive pressure ventilation between induction and laryngoscopy could decrease the incidence of hypoxemia during intubation was uncertain, as was whether positive pressure ventilation would cause gastric insufflation and aspiration. The recent PreVent (Preventing Hypoxemia with Manual Ventilation during Endotracheal Intubation) trial compared bag mask ventilation versus no ventilation between induction and laryngoscopy among 401 critically ill adults undergoing tracheal intubation in seven ICUs in the United States (23). The trial found that bag mask ventilation reduced the incidence of hypoxemia from 22.8% to 10.9% (absolute difference, −12.0 percentage points; 95% CI, −19.3 to −4.6). Aspiration occurred in 2.5% of patients in the bag mask ventilation group versus 4.0% of patients in the no-ventilation group. Together, the results of the PreVent trial, PREOXI trial, and other recent trials (13, 14) suggest that critically ill adults without a specific contraindication to positive pressure ventilation should receive supplemental oxygen and positive pressure ventilation from induction of anesthesia until the initiation of laryngoscopy.

### During Laryngoscopy and Intubation of the Trachea

During laryngoscopy and insertion of an endotracheal tube, positive pressure ventilation cannot be administered, but supplemental oxygen may still be administered via a standard nasal cannula or a high-flow nasal cannula, an intervention referred to as “apneic oxygenation” (23–25). The rationale for administering supplemental oxygen during laryngoscopy and intubation is that although the patient is apneic and gas is no longer moving in and out of the lungs *en masse*, some amount of oxygen administered to the nasopharynx may still make its way to the alveoli through diffusion (24). Among 356 ED and ICU patients undergoing tracheal intubation in two randomized trials, administration of 15 liters per minute of supplemental oxygen by standard nasal cannula between induction of anesthesia and intubation of the trachea did not affect the lowest oxygen saturation or the incidence of hypoxemia during intubation (25, 26). Similarly, among 356 ICU patients undergoing tracheal intubation in three randomized trials, administration of 60 liters per minute of 100% oxygen by high-flow nasal cannula from the beginning of preoxygenation through intubation did not affect the lowest oxygen saturation during intubation (15–17). Although some meta-analyses have suggested that supplemental oxygen during laryngoscopy and intubation may increase the lowest oxygen saturation by a small margin (27, 28), any effect, if present, appears to be much smaller than the beneficial effects of preoxygenation with noninvasive ventilation and positive pressure ventilation between induction and laryngoscopy in an unselected patient population.

## Medications

At the end of preoxygenation, critically ill adults undergoing intubation in the ED or ICU without an indication for awake intubation commonly receive *1*) a sedative medication to induce anesthesia, *2*) a neuromuscular blocking agent to relax the muscles of the head and neck, and *3*) an intravenous fluid bolus or vasopressor to prevent hypotension during intubation (Figure 1).

### Induction Medication

The administration of a sedative medication to induce anesthesia both facilitates the performance of laryngoscopy and intubation by the clinician and aims to ensure that the patient is unconscious and not experiencing discomfort from the procedure. Which induction medication patients receive for emergency tracheal intubation varies significantly by region and provider specialty (2, 29). Etomidate and ketamine are the most common induction medications in many settings in the United States (12, 29–33). Because observational studies have reported propofol to be associated with higher rates of hypotension (34), recent research has focused on which of the induction medications associated with less hemodynamic instability (e.g., etomidate vs. ketamine) results in better patient outcomes. Etomidate has predictable dosing, rapid onset, a short duration of action, and excellent hemodynamic stability but blocks 11-β-hydroxylase in the adrenal glands, inhibiting cortisol production and causing adrenal insufficiency for up to 3 days (35–39). Whether the adrenal insufficiency caused by etomidate affects survival for critically ill adults remains uncertain. Numerous observational studies have reported receipt of etomidate to be associated with increased mortality (29, 35, 40). Several randomized trials have compared ketamine and etomidate for emergency tracheal intubation (41–47). Among 469 critically ill adults undergoing tracheal intubation in the KETASED (Ketamine versus Etomidate during Rapid Sequence Intubation) trial, the primary outcome of the maximum Sequential Organ Failure Assessment score during the first 3 days after intubation was a mean of 9.6 in the ketamine group and 10.3 in the etomidate group (difference, −0.7; 95% CI, −1.4 to 0.0; *P* = 0.056) (41). The incidence of 28-day mortality was 31% in the ketamine group and 35% in the etomidate group (absolute difference, −4 percentage points; 95% CI, −12 to 4). Among 801 critically ill adults in the single-center EvK trial (Etomidate versus Ketamine for Emergency Endotracheal Intubation: a Prospective Randomized Clinical Trial), death by 7 days occurred in 14.9% in the ketamine group and 22.7% in the etomidate group (absolute difference, −7.8 percentage points; 95% CI, −13.0 to −2.4; *P* = 0.005) (42). Currently, experts disagree about whether the choice of induction medication during emergency tracheal intubation affects patient-centered outcomes (30, 47). To address the remaining uncertainty about whether ketamine or etomidate results in better patient-centered outcomes, an ongoing multicenter randomized trial is comparing ketamine with etomidate with regard to 28-day mortality, survival to 12 months, and symptoms of post-traumatic stress disorder among 2,364 critically ill adults in 14 EDs or ICUs (NCT04349501).

### Neuromuscular Blocking Agent

For many critically ill adults undergoing emergency tracheal intubation in current clinical care, the administration of a sedative medication to induce anesthesia is accompanied by the administration of a neuromuscular blocking agent (“paralytic”). However, this practice is not universal, and, in some EDs and ICUs, tracheal intubation is routinely performed without a neuromuscular blocking agent (34, 48–51).

Administration of a neuromuscular blocking agent is intended to relax the muscles of the head and neck to facilitate laryngoscopy and prevent movement of the vocal cords to facilitate placement of an endotracheal tube in the trachea. The potential benefits of administration of a neuromuscular blocking agent include increasing the likelihood of successful intubation on the first attempt and facilitating the ease of bag mask ventilation. The potential risks of administration of a neuromuscular blocking agent include the risks of the medication itself (e.g., hyperkalemia with succinylcholine) and the risks of paralysis in a critically ill adult (e.g., potentially greater risk of hypoxemia during intubation because of apnea) (52–54). The relative risks and benefits of administration of a neuromuscular blocking agent during emergency tracheal intubation have never been examined in a randomized trial. However, a recent 1,150-patient randomized trial of patients undergoing tracheal intubation in the operating room suggested that neuromuscular blockade improved successful intubation on the first attempt (93.5% vs. 88.5%). Although these results may not generalize directly to intubations in the ED and ICU—most patients in the trial were intubated with a direct laryngoscope and hypoxemia was rare—until data from randomized trials in the ED and ICU setting are available, routine administration of a neuromuscular blocking agent for most tracheal intubations in these settings is reasonable.

The two neuromuscular blocking agents most commonly used for tracheal intubation in the ED or ICU are succinylcholine and rocuronium (55, 56). Succinylcholine’s short duration of action of 6 to 10 minutes allows faster resumption of spontaneous breathing. However, succinylcholine may cause rare but serious side effects, including rhabdomyolysis, malignant hyperthermia, and hyperkalemia (54, 57, 58). Rocuronium avoids these rare side effects but has a longer duration of action of 30 to 90 minutes, and recent observational studies have reported an association between receipt of rocuronium and patients experiencing the sensation of being awake but unable to move (“awareness with paralysis” or “awake immobility”) (59, 60). Medications capable of reversing the neuromuscular blockade of rocuronium, such as sugammadex, are now available in some settings. Among 1,248 patients being intubated in the prehospital setting in the CURASMUR (Succinylcholine versus Rocuronium for Out-of-Hospital Emergency Intubation) trial, the incidence of successful intubation on the first attempt was 79.4% in the succinylcholine group and 74.6% in the rocuronium group (61). However, the incidences of arrhythmia, hypotension, and vasopressor receipt were greater in the succinylcholine group. A single-center trial found no difference between succinylcholine and rocuronium in the incidence of desaturation among ICU patients (52). Despite the lack of rigorous evidence to inform which neuromuscular blockade agent results in the best outcomes for patients, over the last decade, rocuronium has replaced succinylcholine as the most commonly used neuromuscular blockade agent in many ED and ICU settings (10, 11). Further randomized trials could compare the effects of succinylcholine versus rocuronium on successful intubation on the first attempt, complications during intubation, awareness with paralysis, and symptoms of post-traumatic stress disorder.

### Interventions to Prevent Hypotension

Hypotension occurs during 25–40% of emergency tracheal intubations and may be associated with an increased risk of cardiac arrest and death (2, 9, 62). Physiological mechanisms that contribute to the development of hypotension during tracheal intubation of critically ill adults include *1*) vasodilation from induction medications, *2*) decreased endogenous catecholamine release after sedation, and *3*) decreased venous return and cardiac output from the initiation of positive pressure ventilation. In addition to ongoing treatments critically ill adults may be receiving for existing hypotension, the two treatments most commonly proposed to prevent the development of hypotension during emergency tracheal intubation are the administration of an intravenous fluid bolus or the administration of vasopressors before or with induction.

Two randomized trials have examined whether the initiation of a 500-ml bolus of intravenous crystalloid solution before induction of anesthesia prevents hypotension during tracheal intubation of critically ill adults. The PrePARE (Preventing Cardiovascular Collapse with Administration of Fluid Resuscitation before Tracheal Intubation) trial examined 337 critically ill adults undergoing tracheal intubation in nine EDs or ICUs, and the PREPARE II (Preventing Cardiovascular Collapse with Administration of Fluid Resuscitation during Induction and Intubation) trial examined 1,067 critically ill adults undergoing tracheal intubation with positive pressure ventilation in 11 ICUs. In both trials, administration of a fluid bolus did not prevent hypotension, vasopressor receipt, or cardiac arrest.

The administration of vasopressors before or with induction of anesthesia is a commonly recommended alternative for attempting to prevent hypotension during tracheal intubation. Such “prophylactic” vasopressors may be administered as either *1*) an intravenous bolus (“push”) or *2*) a continuous infusion. No completed randomized trials have examined whether the administration of a vasopressor before induction of anesthesia prevents hypotension or cardiac arrest during tracheal intubation in the ED or ICU. A propensity score–matched analysis reported that the prophylactic administration of vasopressors before induction was not associated with a lower incidence of hypotension during intubation (40.6% vs. 32.1%; *P* = 0.08) (63). Moreover, multiple observational studies have reported the potential for serious dosing errors with the use of intravenous boluses of vasopressors before emergency tracheal intubation (64, 65). To address this uncertainty, an ongoing multicenter randomized trial is comparing the effect of prophylactic norepinephrine infusion with no vasopressor administration on the incidence of hypotension or cardiac arrest among 420 critically ill adults undergoing tracheal intubation (NCT05014581).

## Laryngoscopy and Tracheal Intubation

After a patient has received preoxygenation and medications for induction of anesthesia, a clinician confirms that the patient is positioned for the procedure (66), obtains a view of the vocal cords (laryngoscopy), and inserts the endotracheal tube between the vocal cords and into the trachea (intubation). Decisions regarding the devices used for laryngoscopy and intubation affect the likelihood of successful intubation on the first attempt (Figure 1).

### Laryngoscope

**Figure 2.**
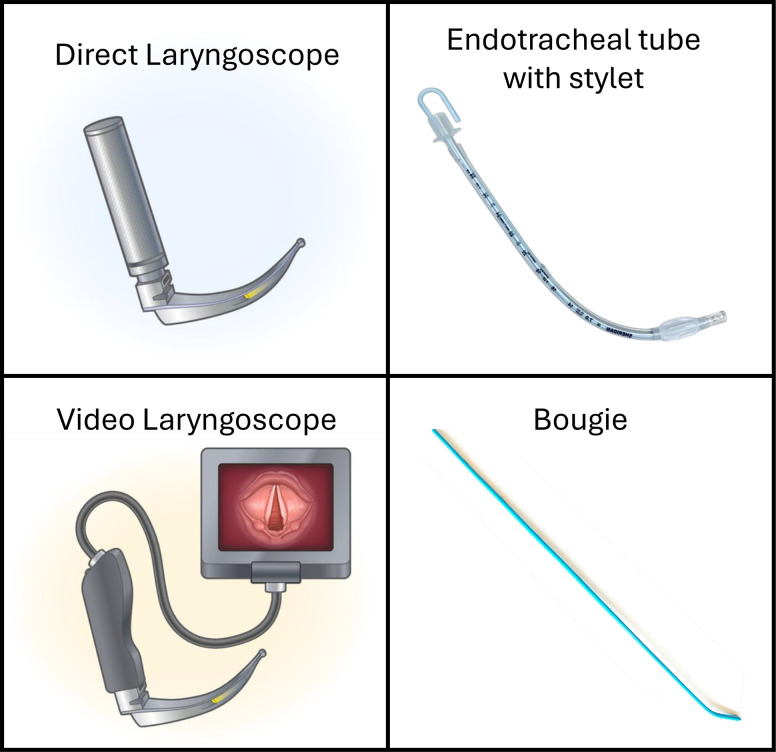
Equipment used for laryngoscopy and intubation of the trachea. The figure shows a direct laryngoscope (upper left panel), a video laryngoscope (lower left panel), and endotracheal tube containing a flexible metal stylet (upper right panel) and an endotracheal tube introducer or gum elastic bougie (lower right panel). The images of the laryngoscopes were adapted from Reference 10. Copyright 2023 Massachusetts Medical Society. Reprinted with permission. The image of an endotracheal tube was adapted from Reference 76 with the following license: https://creativecommons.org/licenses/by/4.0/.

Two types of laryngoscopes are commonly used to perform emergency tracheal intubation: a direct laryngoscope and a video laryngoscope. A direct laryngoscope consists of a handle, a blade, and a light (Figure 2). A video laryngoscope has the same components but also contains a camera near the tip of the blade that transmits images to a screen (67). At least 20 randomized trials have compared video versus direct laryngoscopy for tracheal intubation of critically ill adults (68). The largest of these trials, the DEVICE (Direct versus Video Laryngoscope) trial, found that among 1,417 critically ill adults undergoing tracheal intubation in 17 EDs and ICUs, the incidence of successful intubation on the first attempt was 85.1% in the video laryngoscope group and 70.8% in the direct laryngoscope group (absolute difference, 14.3%; 95% CI, 9.9 to 18.7; *P* < 0.001) (10). Use of a video laryngoscope appeared to be beneficial in every subgroup, and the effect was largest for intubations performed by clinicians with the least prior intubating experience. A recent meta-analysis of randomized trials found that use of a video laryngoscope probably leads to a higher rate of successful intubation on the first attempt (relative risk, 1.13; 95% CI, 1.06–1.21; moderate certainty) and a lower rate of esophageal intubation (relative risk, 0.47; 95% CI, 0.27–0.82; moderate certainty). Except for the purpose of training in direct laryngoscopy or in settings where a video laryngoscope is unavailable, a video laryngoscope should be the primary device used for laryngoscopy during tracheal intubation of critically ill adults.

Whether the shape of the video laryngoscope blade affects the incidence of successful intubation on the first attempt during tracheal intubation of critically ill adults is unknown. Standard geometry video laryngoscope blades approximate the shape of a Macintosh-style curved direct laryngoscope blade and, as with use of a direct laryngoscope, require anterior displacement of the jaw to elevate the epiglottis and expose the vocal cords. Hyperangulated video laryngoscope blades have a more acutely angled shape with a goal of allowing visualization of the vocal cords without significant manipulation of the jaw, particularly for patients anticipated to have an anatomically difficult airway. No multicenter randomized trials have compared standard geometry versus hyperangulated video laryngoscope blades for emergency tracheal intubation. An ongoing randomized trial is comparing standard geometry versus hyperangulated video laryngoscope blades among 1,036 ICU patients undergoing tracheal intubation in Spain (NCT06322719).

### Device for Intubating the Trachea

To insert the endotracheal tube between the vocal cords and into the trachea, a clinician may use an endotracheal tube alone, an endotracheal tube with an intubating stylet, or a gum elastic bougie over which an endotracheal tube is advanced. An intubating stylet is a metal rod (either rigid or malleable) that is placed inside the endotracheal tube to facilitate control of the tube as it advances through the mouth and pharynx to the vocal cords. A gum elastic bougie (hereafter referred to as a “bougie”) is a thin plastic rod that the clinician passes through the mouth and pharynx and between the vocal cords into the trachea, over which the endotracheal tube is then inserted.

The STYLETO (Stylet for Orotracheal Intubation) randomized trial compared intubation using an endotracheal tube plus stylet versus an endotracheal tube alone among 999 critically ill adults in 32 ICUs in France. The primary outcome of successful intubation on the first attempt occurred in 78.2% of patients in the endotracheal tube plus stylet group versus 71.5% of patients in the endotracheal tube alone group (absolute difference, 6.7 percentage points; 95% CI, 1.4 to 12.1; *P* = 0.01). The results of this trial indicate that an endotracheal tube alone generally should not be used during intubation of critically ill adults.

Two randomized trials have compared emergency tracheal intubation using a bougie with intubation using an endotracheal tube with stylet. The BEAM (Bougie Use in Emergency Airway Management) trial found that among 757 patients undergoing tracheal intubation in an ED at which most intubations historically were performed using a bougie, successful intubation on the first attempt occurred in 98% of patients in the bougie group and 87% of patients in the endotracheal tube with stylet group (absolute difference, 11 percentage points; 95% CI, 7 to 14). The BOUGIE (Bougie or Stylet in Patients Undergoing Intubation Emergently) trial found that among 1,102 patients undergoing tracheal intubation in 15 EDs or ICUs in which most intubations historically were performed using an endotracheal tube with stylet, successful intubation on the first attempt occurred in 80.4% of patients in the bougie group and 83.0% of patients in the stylet group (absolute difference, −2.6 percentage points; 95% CI, −7.3 to 2.2). Together, the results of these trials indicate that use of a bougie or use of an endotracheal tube with stylet are both reasonable initial approaches to emergency tracheal intubation and that use of a bougie may safely be reserved for cases in which the operator’s view of the vocal cords is limited.

Little is known regarding the size of the endotracheal tube that should be placed during emergency intubation. Up to half of ICU patients receiving mechanical ventilation develop injury to the vocal cords (69). This injury may lead to irreversible scarring, affecting patients’ ability to breathe, speak, and swallow (70). Observational studies have reported an association between larger endotracheal tubes (i.e., an internal diameter of 8.0 mm for taller patients and 7.5 mm for shorter patients) and injury to the vocal cords. Use of smaller endotracheal tubes (i.e., an internal diameter of 7.0 mm for taller patients and 6.5 mm for shorter patients) might reduce the risk of vocal cord injury but might increase the resistance to gas flow and impede mucus clearance, prolonging the duration of mechanical ventilation. No randomized trials have compared breathing tube size for critically ill adults, and such a trial is urgently needed.

## How to Perform an Evidence-based Tracheal Intubation of a Critically Ill Adult

The approach to emergency tracheal intubation of a critically ill adult in the ED or ICU must be tailored to the patient, clinician, and context. Based on the available evidence from randomized trials (Table 1), the following represents a reasonable standard approach to tracheal intubation of a critically ill adult in the ED or ICU without an anticipated anatomically difficult airway for whom use of sedation and neuromuscular blockade is planned:

- Unless a contraindication to noninvasive ventilation is present, administer noninvasive ventilation through either a dedicated bilevel positive inspiratory pressure machine or a tight-fitting mask connected to a conventional invasive mechanical ventilator beginning 3–5 minutes before induction of anesthesia and ending at the initiation of laryngoscopy (settings, Fi_O_2__ of 100%, inspiratory pressure ⩾10 cm H_2_O, expiratory pressure ⩾5 cm H_2_O, respiratory rate ⩾10 breaths per minute).
- In addition to the general management of shock and resuscitation in critically ill patients, for patients at risk of hypotension during intubation, it may be reasonable for clinicians to choose to initiate a vasopressor infusion or administer a bolus dose of vasopressor before or with the induction of anesthesia or to have these medications available to treat hypotension if it occurs after induction of anesthesia.
- Administer either ketamine or etomidate for induction of anesthesia and either rocuronium or succinylcholine (if no contraindications are present) for neuromuscular blockade.
- With the patient in a sniffing position, perform laryngoscopy using a video laryngoscope with either a standard geometry or hyperangulated blade.
- While viewing the video screen, use either an endotracheal tube with stylet or a bougie to place an endotracheal tube in the trachea.
- Use either colorimetric end-tidal carbon dioxide monitoring or waveform capnography plus auscultation to confirm placement in the trachea.

**Table 1.** Summary of Evidence from Randomized Trials on Emergency Tracheal Intubation

Evidence	Treatment
Evidence of benefit	Preoxygenation with noninvasive ventilation decreases the incidence of hypoxemia during intubation compared with an oxygen mask (12–14). Use of a videolaryngoscope increases the incidence of successful intubation on the first attempt compared with a direct laryngoscope (10, 68). Positive pressure ventilation between induction of anesthesia and initiation of laryngoscopy prevents hypoxemia during intubation (23). Use of a stylet increases the incidence of successful intubation on the first attempt compared with use of an endotracheal tube alone (71). Laryngoscopy and intubation with the patient in the sniffing position may increase the incidence of successful intubation on the first attempt compared with the ramped position (66).
Evidence of lack of benefit	Administration of an intravenous fluid bolus beginning before induction does not prevent hypotension or cardiac arrest during intubation (72, 73). Using a bougie versus an endotracheal tube with stylet may not significantly affect the incidence of successful intubation on the first attempt (74, 75).
More evidence required	Does use of ketamine for induction of anesthesia decrease the incidence of death compared with etomidate? Does use of propofol increase the incidence of hypotension and cardiac arrest compared with ketamine or etomidate? Does use of succinylcholine versus rocuronium affect the incidences of successful intubation on the first attempt, complications during intubation, awareness with paralysis, or post-traumatic stress disorder? Does intubation with versus without neuromuscular blockade affect the incidences of successful intubation on the first attempt, complications during intubation, awareness with paralysis, or post-traumatic stress disorder? Does the order of medication administration (sedative first vs. neuromuscular blocking agent first) affect the incidences of successful intubation on the first attempt, complications during intubation, awareness with paralysis, or post-traumatic stress disorder? Does administering ketamine to facilitate preoxygenation before induction of anesthesia and neuromuscular blockade (“delayed sequence intubation”) facilitate successful intubation on the first attempt and decrease the incidence of complications? Does administration of a vasopressor before induction of anesthesia prevent hypotension and cardiac arrest during intubation? Does administration of supplemental oxygen by standard nasal cannula or high-flow nasal cannula between induction of anesthesia and intubation of the trachea prevent hypoxemia during intubation? Does use of a hyperangulated versus standard geometry videolaryngoscope blade affect the incidence of successful intubation on the first attempt? Does use of a smaller endotracheal tube prevent problems with breathing, speaking, and swallowing without increasing the duration of mechanical ventilation compared with a larger endotracheal tube?

This table summarizes treatment decisions that clinicians make during each tracheal intubation in an emergency department or ICU for which evidence from randomized trials demonstrates a benefit, demonstrates no difference, or is insufficient to inform care.

Beyond the treatment choices for which rigorous evidence now exists to inform care, clinicians must make numerous other treatment decisions during emergency tracheal intubation for which evidence is currently lacking (Table 1). Generating rigorous evidence to inform these knowledge gaps represents an important opportunity to continue to improve the safety of this common and high-risk procedure for critically ill adults.

## Conclusions

In the last two decades, a series of randomized trials have identified treatments that improve outcomes of this high-risk procedure. Many of the most important decisions clinicians make during emergency tracheal intubation, however, are not yet informed by rigorous evidence. Systematically conducting randomized trials to examine every aspect of this common and high-risk procedure is improving procedural and patient outcomes and has the potential to bring forth an era of evidence-based emergency tracheal intubation.
